# The effect of surgical complications on trauma and orthopaedic trainees

**DOI:** 10.1308/rcsann.2025.0119

**Published:** 2026-01-20

**Authors:** A Faraj, J Battle, J O’Callaghan, Trauma and Orthopaedic Trainee Collaborators

**Affiliations:** Gloucestershire Royal Hospital, UK

**Keywords:** Complications, Trainees, Trauma, Orthopaedics

## Abstract

**Background:**

Complications can be devastating for patients, but the ‘second victim’ phenomenon is increasingly being acknowledged, whereby the surgeons involved in the complication are adversely affected by such complications. For trainees, who are early into their surgical careers and are on a learning curve, such events can be formative or pivotal in their careers. Additionally, as temporary rotating members of the departments they work in, there can often be educational, interpersonal or workplace demands that amplify such effects, and a lack of professional ties that allow them to comfortably discuss complications with temporary or new work colleagues.

**Methods:**

An online questionnaire was designed and distributed to UK Trauma and Orthopaedic trainees. Sixty-five trainees responded from across ten deaneries.

**Results:**

There were significant negative effects of complications on trainees, including sadness (77.8%), anxiety (63.5%), guilt (69.8%) and embarrassment (63.5%). A total of 40.3% reported complications affected them outside of work. Only 60.9% felt well supported. Only 22.2% were offered formal support. In addition, 66.7% have witnessed another trainee struggle after a complication. Only 15.6% felt their training programme adequately prepares them to deal with the emotional impact of complications.

**Conclusions:**

Adverse effects of complications on Trauma and Orthopaedic trainees are a prevalent issue. There are no ubiquitous formal structures in place to support trainees affected by complications. Nonblame, informal debrief sessions were cited repeatedly as beneficial.

## Introduction

Surgical complications are an inherent risk in operative care, representing a critical challenge in both patient safety and surgical education.^1,2^ For surgical trainees—who are often still developing their clinical judgment, technical competence and professional identity—these complications carry profound implications.^3,4^ This is particularly relevant in orthopaedic surgery—a specialty recognised for its high volume and technical challenge, with potential for adverse outcomes.^5,6^ These can range from minor intraoperative issues to major postoperative failures. Given these wide-ranging complications, safely managing them is one of the key curriculum-based objectives for UK orthopaedic trainees.^7^ Experiencing complications during training not only affects patient outcomes but can also shape the trainee’s confidence, learning trajectory and emotional wellbeing.^8,9^

The psychological impact of surgical complications and medical errors on healthcare providers has been increasingly recognised. The concept of the ‘second victim’, coined by Wu,^10^ refers to the emotional distress experienced by clinicians involved in adverse events. This phenomenon can manifest as guilt, anxiety, loss of self-confidence and even symptoms resembling post-traumatic stress disorder.^8^ Among trainees, these responses may be intensified due to limited experience, uncertainty about clinical decisions and hierarchical workplace structures.^11^ In surgical specialties, where complications often result in visible and immediate patient harm, the emotional burden is particularly pronounced.^12^

Studies have shown that trainees frequently treat complications as personal failures rather than system failures, particularly when responses to failures focus on blame rather than learning.^13^ A prevailing culture of silence around surgical errors may discourage open discussion and hinder opportunities for reflection and improvement.^14^ Furthermore, fear of litigation or professional repercussions can hinder the disclosure of errors and limit learning from them.^15^

Nevertheless, complications can serve as powerful educational tools when managed within a supportive and structured framework that promotes reflection, mentorship and feedback. Evidence from teaching hospitals supports this approach, demonstrating improved patient outcomes and reduced mortality when complex surgical procedures are performed in educational environments.^16–18^ Kolb’s theory highlights the importance of reflective observation and abstract conceptualisation in transforming experience into knowledge.^19^ However, the educational benefit of complications is highly dependent on the learning environment. Psychological safety—the shared belief that one can speak up and take risks without fear of punishment—is essential for fostering open discussion and critical reflection.^13,20^

The impact of complications also goes hand in hand with trainee wellbeing. Burnout among surgical trainees is common and has been linked to emotional exhaustion, depersonalisation and reduced sense of personal accomplishment.^21^ Complications may act as acute stressors that exacerbate burnout, particularly when support mechanisms are inadequate. Previous studies have shown that trainees often lack formal structures for emotional support following complications, relying instead on informal peer networks or personal coping strategies.^22,23^ Despite this, evidence specific to orthopaedic trainees remains limited, representing an important gap in understanding.

This study aims to explore the effects of surgical complications on orthopaedic trainees in the UK at various stages of their training. This study aims to investigate through questionnaire feedback how trainees interpret and reflect on errors and whether their workplace provides a safe learning environment. The aim of the study was to inform strategies for improving the understanding of how trainees deal with mistakes and how they are supported.

## Methods

The study was conducted using an online survey tool (Google Forms). A 29-question survey was distributed to orthopaedic trainees in May 2025. The questionnaire was constructed based on a preceding literature review that identified core themes for trainees experiencing complications.

The questionnaire was split into five distinct parts, addressing: (1) trainee demographics (deanery, year of training, age, gender); (2) descriptions of complications; (3) impact of complications on trainees, using a 1–5 point Likert scale from strongly disagree to strongly agree; (4) support offered to trainees and the opinions on what more could be offered to trainees; (5) final comments and white boxes for further information.

Subgroup analysis based on gender, age group and training grade was performed by converting the Likert scale answers to a numerical score of 1 (strongly disagree) to 5 (strongly agree). Average scores to all questions in Section 3 were then calculated for each respondent. Mean and standard deviation was calculated for each subgroup, and statistical analysis performed to compare genders (unpaired *t*-test), age group (analysis of variance (ANOVA)), and training grade (ANOVA).

White space questions were analysed independently by two different reviewers before they agreed on key themes.

## Results

Section 1 explored trainee demographics. A total of 65 trainees responded to the survey, of which 63 reported experiencing a surgical complication (96.9%). Data were collected from ten deaneries: East Midlands, Kent Surrey Sussex, South East London, Scotland, Severn, Peninsula, Thames Valley, Wessex, West Midlands, Yorkshire and the Humber. All trainee grades from ST3 to ST8 were represented; 47 respondents were male (72.3%) and 18 were female (27.7%).

Section 2 explored the types of complications. Participants were asked to describe the complication that had affected them most in their careers. We have grouped and summarised these complications in Table 1 below.

**Table 1:** Types of complication described by respondents

Types of complication	*n*
Intra-operative:	
Malreduction of fracture or malalignment of arthroplasty	8
Cause or propagating fractures	6
Screw malposition	3
Cement issue	2
Damage to surrounding structures	12
Medical complication	3
Postoperative:	
Postoperative instability/refracture/failure of metalwork/DHS cut out	9
Dislocation of arthroplasty	6
Infection	3

DHS = dynamic hip screw

Section 3 explored the impact of complications on trainees: 77.8% felt responsible (agree or strongly agree); 87.3% felt sadness (agree or strongly agree); 63.5% felt anxious or worried (agree or strongly agree); 17.5% felt anger (agree or strongly agree); 69.8% felt guilty (agree or strongly agree); 63.5% felt shame/embarrassment (agree or strongly agree); 53.2% felt it affected their confidence (agree or strongly agree); 15.9% avoided similar cases for a time (agree or strongly agree); 40.3% felt it affected them outside of work (agree or strongly agree) and 9.5% considered changing specialty or leaving medicine (agree or strongly agree).

Subgroup analysis of section 3 responses are displayed in Table 2, and found little difference between impact on trainees based on their gender, age group or training grade. No significant difference was found between subgroups.

**Table 2:** Likert scale mean response to all questions combined, divided by subgroup

Subgroup	All questions combined			*p*
	n	Mean	±Standard deviation	
Gender				0.272
Male	46	3.27	1.17	
Female	17	3.11	0.87	
Age group				0.603
26–30	9	3.07	0.69	
31–35	41	3.19	0.93	
36 or over	13	3.45	1.18	
Training grade				0.150
ST3	13	3.33	0.72	
ST4	9	2.62	1.13	
ST5	15	3.48	0.75	
ST6	8	3.66	1.01	
ST7	6	3.06	1.17	
ST8	7	3.57	0.76	

Section 4 explored the support offered for trainees: 60.9% of respondents said they felt well supported, 25.0% felt partially supported and 6.3% felt unsupported. The majority (84.1%) debriefed with a consultant, 33.3% with a fellow registrar, 63.5% with another colleague, 34.9% with family and 20.6% with friends. Only 22.2% were offered formal support, with 58.7% not offered formal support afterwards and 19.0% not aware of formal support structures.

Finally, section 5 found that 66.7% have witnessed another trainee struggle after a complication. Only 15.6% felt their training programme adequately prepares them to deal with emotional impact of complications (agree or strongly agree); 57.8% would find structured sessions helpful in dealing with future complications.

Responses to the white space question: ‘Do you have any suggestions on how to better support orthopaedic trainees after a complication?’ are summarised in Table 3 with key themes identified. Trainees are asking for structured debriefs, peer support, a genuinely no-blame culture, active consultant mentorship and protected learning spaces. They also desired emotional support, resilience training and higher-quality training environments to reduce the likelihood of complications and the distress that follows.

**Table 3:** Themes of trainee suggestions on how better to support trainees after a complication

Theme	What trainees suggested	Examples from responses
1. Debriefing as essential support	Structured, timely debriefs (both immediate ‘hot’ and later ‘cold’), ideally consultant-led but supportive. Clear pathways for access.	‘Debrief after event essential’ / ‘Hot debrief always, supportive environment’ / ‘Make a definite standard pathway that the trainee can access acutely’
2. Peer support and shared experiences	Opportunities to share experiences with peers (locally, deanery-wide).	‘Talk to colleagues to realise you’re not alone’ / ‘Sharing stories of complications is helpful’
3. Supportive culture and no-blame environment	Promote openness and honesty, remove blame and shaming. Model normalisation of complications.	‘Frank debrief in departmental meeting with truly no-blame culture’ / ‘Bullying / shaming exists to an extent’ / ‘More openness about complications—we could all learn from each other’
4. Consultant and mentor involvement	Consultants and mentors to provide constructive support, be approachable and trained in supporting trainees. Expand mentoring schemes.	‘Debriefing by a senior team member’ / ‘Support from consultants’
5. Formalised learning opportunities	Structured sessions at deanery/departmental level. Consultants sharing their own experiences and coping strategies.	‘Regular trainee-led meetings to discuss complications’ / ‘Talks from consultants with experiences and coping strategies’ / ‘Dedicated sessions on consultants discussing personal experiences’
6. Emotional and psychological support	Access to counselling, resilience training, signposting of resources by seniors. Validation of emotional impact.	‘Counselling available—should be suggested by consultant’ / ‘It will be good to have some training sessions on resilience’ / ‘Need to learn to put things behind you but it is incredibly difficult’
7. Improving training and operating environment	Better quality training, more consultant time for supervision, less pressured environments.	‘Better training and more time allocated for consultants to train registrars’ / ‘See one-do one-teach one doesn’t prepare you’ / ‘Less stressful operating environment from consultants’ body’

## Discussion

Our findings demonstrate that complications are almost universally experienced by orthopaedic trainees in the UK, with 96.9% of respondents reporting at least one complication during training. While complications are an unavoidable part of surgical practice,^1,2^ they have a profound emotional impact, with guilt, sadness, anxiety and loss of confidence reported by most respondents. These experiences align with the ‘second victim’ phenomenon,^8,10^ in which adverse events trigger cycles of emotional distress and impaired confidence among healthcare professionals.

A striking 69.8% of trainees reported guilt and 63.5% reported shame or embarrassment following a complication. These emotions are intensified in environments where errors are seen as personal failings rather than opportunities for systemic learning.^13,14^ This aligns with previous literature emphasising the necessity of psychological safety in surgical education.^13,20^ Without such safety, open reflection and growth are curtailed, limiting the potential for experiential learning.^19^

We found these emotional responses to complications to affect trainees similarly, regardless of gender, age, or training grade. However, sample sizes in this study are small, and a larger sample size could unmask a statistically significant difference and identify any particularly vulnerable groups.

Despite the prevalence of complications and their impact, only 22.2% of respondents reported being offered formal support, with most relying on consultants, peers or family. Although debriefing with senior colleagues was common, the lack of structured support frameworks is concerning given the high rates of sadness, anxiety and burnout. Previous work suggests that formalised support, including structured debriefs and psychological interventions, can mitigate distress and improve professional resilience.^8,22^ Furthermore, burnout—already prevalent among surgical trainees^21,23^—may be exacerbated by complications, acting as acute stressors in an already high-pressure environment.

Encouragingly, most respondents debriefed with consultants, suggesting that supervisory structures exist to some degree. However, the quality and consistency of these debriefs remain variable. Our results suggest that trainees desire more structured sessions on managing the emotional consequences of complications, with over half (57.8%) indicating such sessions would be beneficial. Integrating these sessions into training curricula would support the inclusion of nontechnical skills, reflective practice and resilience training in surgical education.^20^

From an educational standpoint, complications represent powerful learning opportunities. Reflection is embedded in the Intercollegiate Surgical Curriculum Programme and is a key method of learning and growth. Evidence from teaching hospitals suggests that can improve patient outcomes and lower mortality.^16–18^ However, the educational benefit for trainees depends on supportive learning environments where complications are openly discussed and framed constructively. If neglected, the risk is twofold: erosion of trainee confidence, reported by 53.2% of respondents, and compromised patient safety through avoidance behaviours. The way trainees respond to complications also represents important preparation in how they respond to complications in the future as consultants, as well as how they help future generations of trainees in dealing with complications in turn.

The white space question responses suggest that Orthopaedic trainees value structured and supportive approaches following complications. Debriefing was identified most frequently as essential, both immediately and after the event, with a clear preference for constructive, nonjudgemental feedback. Peer-to-peer discussions were highly regarded for normalising experiences and reducing isolation, reflecting wider evidence on the role of peer support in resilience.^24^ Consultant involvement was considered crucial, with trainees seeking approachable mentors who could provide guidance without blame. This supports existing literature emphasising the importance of senior modelling in fostering a learning culture.^10^

Respondents also called for formalised learning opportunities, such as local or deanery sessions where complications are discussed openly, and senior colleagues sharing their own experiences. Beyond technical aspects, many trainees acknowledged the psychological impact of complications, suggesting benefits from resilience training, counselling and better signposting of support services.^25^ Finally, concerns were raised about the training environment, with requests for greater consultant time dedicated to supervision and reduced pressure in theatre. Overall, the findings highlight that effective support requires attention to educational, emotional and cultural factors.

Our study mirrors recent results from studies of ENT trainees in the UK and orthopaedic trainees in the US,^12,26^ with similar proportions of trainees experiencing complications and those affected emotionally. Moreover, trainees in both studies were most likely to discuss complications initially with a colleague or family member rather than a structured departmental forum, such as Mortality and Morbidity meetings. Our findings also indicate that trainees feel their training programme does not adequately support them in managing complications and that 57.8% would find structured sessions helpful. Together, this provides a strong argument for education programmes to provide formal debriefing sessions emphasising reflection and just culture principles to support learning and emotional processing.^27,28^ These sessions could avoid the inconsistencies and stressful environment often associated with Mortality and Morbidity meetings.^29^

There are several limitations in our study. First, it relies on self-reported data, which may be influenced by recall bias or individual interpretation of ‘complication’. Second, while responses were drawn from ten UK deaneries, the sample size remains modest relative to the national trainee population. Third, qualitative depth is limited compared with interview-based methodologies, although the inclusion of white-box responses partially mitigates this. Finally, the study may suffer from self-selection bias, with trainees who have experienced complications being more likely to participate.

Nonetheless, these findings provide valuable insights into the lived experiences of orthopaedic trainees. They underline the need for systemic change in how complications are addressed in training—shifting from implicit, individualised coping to explicit, programmatic support structures. This aligns with the broader imperative in surgical education to create psychologically safe environments that support resilience, foster reflective learning, and prioritise both trainee wellbeing and patient safety.

## Conclusion

Surgical complications are an unavoidable aspect of orthopaedic practice, but for trainees they represent pivotal moments with both emotional and educational consequences. This study demonstrates that complications are experienced almost universally, with significant impacts on guilt, confidence and wellbeing. Although many trainees receive some level of informal support, formal mechanisms remain inconsistent and underdeveloped.

These findings underscore the need for surgical training programmes to recognise the emotional impact of complications, embed structured debriefing and reflective practice into curricula, and prioritise psychological safety as a foundation for learning. By reframing complications as shared learning opportunities rather than personal failings, training systems can not only protect trainee wellbeing but also improve resilience, patient safety and long-term professional development.

## Artificial Intelligence

The author/s declare that no AI was used to conduct the study or prepare the manuscript.
